# Roles of the Phosphorylation of Transcriptional Factors in Epithelial-Mesenchymal Transition

**DOI:** 10.1155/2019/5810465

**Published:** 2019-06-02

**Authors:** Rong Xu, Jae-Yeon Won, Chang-Hyeon Kim, Da-Eun Kim, Hyungshin Yim

**Affiliations:** Department of Pharmacy, College of Pharmacy, Institute of Pharmaceutical Science and Technology, Hanyang University, Ansan, Gyeonggi-do, Republic of Korea

## Abstract

Epithelial-to-mesenchymal transition (EMT) is the first step in the development of the invasive and migratory properties of cancer metastasis. Since the transcriptional reprogramming of a number of genes occurs in EMT, the regulation of EMT transcription factors has been intensively investigated. EMT transcriptional factors are commonly classified by the direct or indirect repression of E-cadherin because one of hallmarks of EMT is the loss of E-cadherin. This facilitates the expression of genes for EMT, tumor invasion, and metastasis. The posttranslational modification of EMT transcriptional factors, such as Snail and Slug, directly regulates their functions, including their stability, nuclear localization, protein-protein interaction, and ubiquitination for the promotion or termination of EMT at the specific points. Here, we discuss how posttranslational modifications regulate gene expression in a dynamic and reversible manner by modifying upstream signaling pathways, focusing in particular on the posttranslational modifications of Snail, Slug, ZEB1, ZEB2, and TWIST1. This review demonstrates that EMT transcription factors regulate metastasis through their posttranslational modifications and that the flexibility and reversibility of EMT can be modified by phosphorylation.

## 1. Introduction

Cancer metastasis begins with the migration and invasion of cancer cells to the surrounding tissues and involves the loss of cell-cell adhesion. For this, cancer cells acquire mesenchymal characteristics by altering the levels of genetic expression during the epithelial-to-mesenchymal transition (EMT). This alteration of gene expression increases cell migration and invasion during EMT, such that EMT is considered as the first step of cancer metastasis. EMT is driven by various cytokines and growth factors, including transforming growth factor-*β* (TGF-*β*), Wnt, Notch, EGF, FGF, and HGF [1]. Wnt signals generate the translocation of *β*-catenin to the nucleus, which triggers the transcriptional activation of TCF/LCF1 transcriptional complex. This activation is also generated by TGF-*β* signals in cancer cells through the activation of Smad protein by phosphorylation [2]. The TGF-*β* signals crosstalk with Wnt, Notch, and receptor tyrosine kinase signals to induce the specific expression of EMT transcription factor and its functions in cancer metastasis, depending on the cellular context [1]. The treatment of TGF-*β* in cancer cells results in the genetic alteration of important genes through transcriptional regulation, primarily targeting TGFB1 and TGFB3, which are repressed by c-Myc and Oct4/Klf4, respectively [3]. EMT-inducing transcription factors interact with epigenetic regulators to control the expression of genes associated with cell polarity, cell adhesion, the cytoskeleton, and extracellular matrix degradation via the repression of epithelial genes [4].

EMT transcription factors are commonly classified according to the direct or indirect repression of E-cadherin, since a hallmark of EMT is the loss of E-cadherin, which is associated with the acquisition of metastatic activity. Several studies suggest that the posttranslational modification of the E-cadherin gene (*CDH1*), such as* CDH1* silencing, is a critical mechanism for EMT. The* CDH1* promoter contains E-box elements that are responsible for transcriptional repression [5]. The binding of the zinc finger transcription factor Snail to the E-box elements of the* CDH1* promoter has been found to repress the transcription of* CDH1* [5]. Slug, a member of the Snail family [6], as well as ZEB1 [7], and ZEB2 [8] can repress the transcription of* CDH1*, thereby promoting the dissociation of cell adhesion, consequently inducing cell invasion and migration. E47 [9] and KLF8 [10] are also known as direct repressors of the* CDH1* promoter. Although the bHLH factor TWIST1 can induce EMT, it indirectly represses the transcription of* CDH1* [11], which is an indirect repressor of the* CDH1* promoter. Similar to TWIST1, goosecoid [12], FOXC2 [13], SIX1 [14], and bHLH factor E2.2 [15] can suppress the transcription of* CDH1* in a seemingly indirect manner. The activation of these transcription factors drives tumor invasion and metastasis, inducing the transition to mesenchymal characteristics by repressing the transcription of* CDH1* and activation of mesenchymal* CDH2. *

Although these several transcription factors can induce the EMT, their specific functions display in a different way [16, 17]. In melanoma, the difference between ZEB1 and ZEB2 has been studied well [18]. ZEB1 and ZEB2 have different binding corepressors or coactivators based on their structural difference and cell context. Basically ZEB2 is expressed in normal melanocyte, while ZEB1 is highly expressed in melanoma [16, 18]. Like their expression patterns, their functions show an opposing way. ZEB1 cooperates with TWIST1 for their oncogenic properties, while ZEB2 and Slug work together for tumor-suppression [16, 18]. The different expression and functions of transcription factors are connected with the diverse EMT signaling pathways, depending on tissues and context. In addition, the signaling pathways triggered by Wnt, Notch, TGF-*β*, EGF, and several stresses are not linear, but complex and crosstalk. Their differences of expression and functions make the regulation of EMT more precise and tighter.

The activity, specificity, and accuracy of transcription factors are commonly regulated by posttranslational modifications, especially phosphorylation. In response to changes in extracellular and/or intracellular signaling, activated protein kinases such as extracellular signal-regulated kinases and casein kinases in EMT signaling pathway phosphorylate transcription factors and related coregulators, facilitating a program of gene expression. These posttranslational modifications regulate the physiology of transcription factors to induce mesenchymal characteristics while repressing epithelial characteristics. The phosphorylation and dephosphorylation of EMT transcription factors, such as Snail and Slug, can directly regulate their function, including their stability, nuclear localization, protein-protein interaction, and ubiquitination for the termination of their function at a specific time point. In this review, we discuss the mechanisms by which posttranslational modifications dynamically and reversibly regulate gene expression activity by modifying upstream signaling pathways. In particular, this review focuses on the posttranslational modifications of Snail, Slug, ZEB1, ZEB2, and TWIST1, because the factors trigger the classic EMT process through activation of mesenchymal factors and suppression of epithelial factors by regulation of gene expression, as the core EMT transcriptional factors. As the results, invasiveness and migrating activity are acquired in cancer cells [16, 17]. In addition, they are connected with the several characters related with the stemness of cancer stem cell and changing cell metabolism for cell survival [16]. Moreover, their functions are converged on the suppression of E-cadherin, but their regulatory functions for EMT are diverse in the different context with specific manner. This review provides an overview of how these core EMT transcription factors regulate metastasis via posttranslational modifications.

## 2. Snail (Snail1;* SNAI1*)

### 2.1. Function and Structure of Snail

Numerous studies have shown that Snail (product of* SNAI1*), a member of the Snail superfamily of zinc finger transcription factors, functions as a strong inducer of EMT, which converts epithelial cells to mesenchymal cells by the acquisition of migratory and invasive properties through the repression of* CDH1*, switching from* CDH2* expression [5, 47, 48]. Snail has several transcriptional targets for EMT with epithelial factors such as desmoplakin [47] and Muc-1 [49] and mesenchymal factors, including vimentin and fibronectin [47]. The importance of Snail is based on its ability to induce EMT and its positive correlation with malignancy. Its expression is sufficient to induce EMT [47] and is positively correlated with tumors and metastasis [50–52]. Snail is highly expressed in high grade tumors, metastatic cancer, and recurring cancer [50, 51].

**Figure 1:**
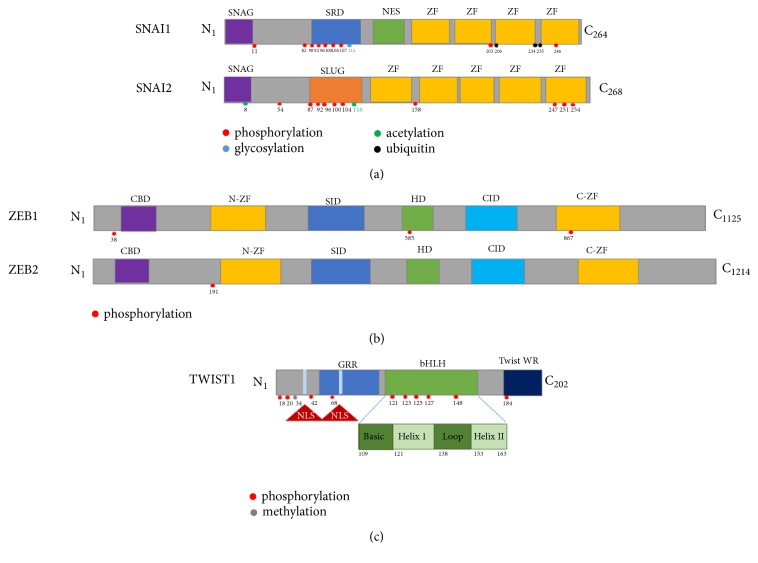
*Structures of the core EMT-TFs*. (a) Snail has N-terminal SNAG domain and C-terminal DNA binding domain of four C2H2 zinc finger (ZF) motifs. Nuclear export sequences (NES) and a serine-rich domain (SRD) are located at the central region of Snail. Slug has N-terminal SNAG domain as a transcriptional repressor, proline-rich SLUG domain in the central region, and C-terminal five zinc finger motifs. (b) ZEB has two zinc finger clusters that are N-terminal zinc finger (NZF) and C-terminal zinc finger (CZF) and a centrally located homeodomain (HD). The corepressors bind to the protein binding domain of ZEB1, which are CtBP interaction domain (CID) at C-terminus, Smad interaction domain (SID) and homeodomain (HD) at the center, and CAF/p300 binding domain (CBD) at N-terminus of ZEB1. (c) Twist1 has a DNA-binding basic region (amino acids 109–121) and a bHLH domain (amino acids 122-163) and Twist WR domain (amino acids 182-202) for the transcriptional activity. SRD, serine-rich domain; ZF, zinc finger domain; NES, nuclear export sequence; CBD, coactivator binding domain; SID, Smad interaction domain; BD, homeodomain; OD, CtBP interaction domain; NLS, nuclear localization signals.

Snail was first identified in* Drosophila melanogaster* [53] and Snail homologues have been detected in several species, from* Drosophila melanogaster* to* Homo sapience*. The Snail superfamily includes Snail (Snail 1), Slug (Snail 2), and Smuc (Snail 3), which share several common structures, with a C-terminal DNA-binding domain and an N-terminal SNAG domain (Figure 1(a)). The SNAG domain, originally defined as a repressor motif in zinc finger proteins, such as Snail and Gfi-1 [54], is critical for the binding of transcriptional corepressor complexes, including histone deacetylase 1/2 [55], 14-3-3 [56], and Ajuba [57]. The C-terminal domain has four zinc fingers and an E-box motif-binding region [58]. Nuclear export sequences (NES) and a serine-rich domain (SRD) are located at the central region of Snail [59] (Figure 1(a)). Detailed studies show that the activity and stability of Snail are regulated by posttranslational modification in several residues of Snail, depending on the extracellular signaling and tumor microenvironment, as discussed below.

### 2.2. Transcriptional Repressor Activity by Posttranslational Modification

The most important function of Snail is the transcriptional repression of the* CDH1* gene, an epithelial marker, by binding to the E-element of the* CDH1* promoter region via its SNAG domain [55]. When the SNAG domain of Snail binds to the* CDH1* promoter region, the recruitment of the mSin3A/HDAC1/2 corepressor complex is required [5, 47, 55]. The phosphorylation of Snail at the S11 residue near the SNAG domain by PKA and at the S92 residue by CK2 increases the efficiency for the recruitment of the mSin3A corepressor, which is required for the repressive activity of* CDH1* (Table 1) [19]. The role of CK2 in EMT is demonstrated by the unbalanced expression of the CK2 subunit, which drives EMT in breast epithelial cells [60] and is related to the modification of Snail. In addition, PAK1, an interacting partner of the motility regulators, GTPase, Rac1, or Cdc42, also phosphorylates Snail at the S246 residue in the zinc finger domain, promoting the transcriptional repression of Snail targeting the E-cadherin and occludin promoters [26]. Based on these studies, the phosphorylation sites of Snail related with repression are not limited to the SANG domain, which is critical for the binding of the transcriptional corepressor, but are also distributed on the SRD and the C-terminal DNA-binding domain. Although the phosphorylation of Snail mostly promotes EMT through E-cadherin suppressive activity, PKD1-mediated phosphorylation of Snail at S11 suppressed EMT and reduced its transcriptional repressive activity [20, 21], which is associated with the epigenetic suppression of PKD1 in several cancers.

### 2.3. Nuclear Accumulation by Posttranslational Modification

Nucleocytoplasmic shuttling is the main mechanism that regulates the spatial-dependent function of transcription factors. The nuclear location is mandatory for the transcriptional regulation of transcription factors. Snail localizes in the nucleus primarily via nuclear localization signals (NLS). Snail has three NLS motifs in the N-terminal region (amino acids 8-16), which overlap with the SNAG domain, and a middle region (amino acids 151-152) that is proximal to the DNA-binding domain [61]. NES is located in the middle region of Snail for export to the cytoplasm [59]. In addition to NLS and NES, nucleocytoplasmic shuttling is also regulated by the phosphorylation of Snail by several kinases. PAK1 phosphorylates Snail at the S246 residue, which modulates its transcriptional activity by increasing its accumulation in the nucleus, which in turn increases the E-cadherin repression activity of Snail [26]. The S246 residue is also phosphorylated by PI3K, induced by growth-regulated protein *α*, which has been found to increase EMT and bladder cancer recurrence [28]. The phosphorylation of Snail at T203 in the nucleus by Lats2, a serine/threonine kinase in mitosis, was found to increase its retention in the nucleus in response to multiple signals, including TGF-*β*-induced EMT [25]. In response to the collagen receptor DDR2, ERK2 is activated and phosphorylates Snail at residues S82 and S104, leading to the nuclear accumulation of Snail and the suppression of E-cadherin expression [22]. Thus the nuclear accumulation induced by phosphorylation is directly connected with the transcriptional activity of Snail and its function.

### 2.4. Ubiquitination and Degradation by Posttranslational Modification

Snail is a highly unstable protein with a short half-life approximately 25 min [62]. Snail has two consensus motifs binding with GSK3*β*, which phosphorylate Snail at S92, S96, S100, and S104, located in the SRD, which regulates its stability [23, 24, 62]. The phosphorylation of the first motif at S96 and S100 regulates its *β*-transducin repeat-containing protein- (*β*-TRCP-) mediated ubiquitination [23, 24]. For GSK3*β*-mediated phosphorylation, Snail is phosphorylated at S104 and S107 by CK1 as a priming site for the subsequent phosphorylation by GSK3*β* [24]. CK1-mediated priming phosphorylation allows for GSK3*β*-mediated phosphorylation of residues S100 and S96, as well as residue S92 as a subsequent phosphorylation reaction. The phosphorylation of residues S96 and S100 demonstrates that *β*-TRCP recognition sites play a role in protein polyubiquitination and degradation [24].

### 2.5. Stabilization of Snail by Posttranslational Modification

The phosphorylation of Snail at residues S82 and S104 by ERK2, residue S246 by PAK1, and residue T203 by Lats2 induced the nuclear retention of Snail and increased its activity. The nuclear localization of Snail by phosphorylation increases its stability as it allows it to escape from *β*-TRCP-mediated polyubiquitination and degradation. For stability, Snail is also monoubiquitinated at K206, K234, and K235 by ubiquitin-editing enzyme A20 [30]. Monoubiquitinated Snail1 has a reduced affinity for GSK3*β*, and thus, Snail is stabilized in the nucleus due to the decreased phosphorylation mediated by GSK3*β* [30]. In addition, the* O*-GlcNAc modification of Snail increases its stability by suppressing protein degradation [29]. The* O*-GlcNAc modification at S112 disrupts the CK1-mediated priming phosphorylation at residues S104 and S107, inhibiting GSK3*β*-mediated degradation [29]. Consequently,* O*-GlcNAc modification stabilizes Snail to avoid the protein degradation and thereby increases its transcriptional repressor activity for* CDH1* expression.

The phosphorylation of Snail can be influenced by environmental conditions. In terms of the functional regulation of Snail by phosphorylation, PKA, CK2, PAK1, PI3K, Lats2, and ERK2 are positive regulators of Snail that support its transcriptional activity, nuclear localization, and stabilization. However, the phosphorylation of Snail by PKD1, GSK3*β*, and CK1 suppresses its transcriptional activity and induces its degradation. Consequently, they function as negative regulators of Snail and EMT. The balance between a positive and negative regulator is thus dependent on the cellular context.

## 3. Slug (Snail2;* SNAI2*)

### 3.1. Function and Structure of Slug

Slug (product of* SNAI2*) is a member of the Snail family and has a zinc finger domain with transcriptional repressor activity. Slug shares common characters with Snail based on its structure. Slug has C-terminal five zinc finger DNA-binding domains with an E-box motif (CAGGTG)-binding region which is required for transcriptional activity as a transcription factor [63, 64]. In the N-terminus, Slug also has a SNAG domain, which acts as a transcriptional repressor (amino acids 1-32) and which is separated from the C-terminal zinc finger domain [64]. Slug has similar NLS as Snail [65]. In spite of the similarities between Snail and Slug, Slug has specific proline-rich domains in the central region, i.e., the SLUG domain, although the function of SLUG domain is uncovered [66]. As Snail binds to mSin3A, a corepressor for* CDH1* repression, Slug also interacts with the corepressors NCoR and CtBP1 to transcriptionally repress* CDH1* [66]. NCoR and CtBP1 are recruited as transcriptional regulators at different Slug binding regions. NCoR binds to Slug through the SLUG domain, whereas CtBP1 is recruited to the SNAG domain [66]. Depending on the specific cellular conditions and environmental context, the expression of corepressors, such as NCoR, CtBP1, and mSin3A, may differ, which determines the dominant role of either Snail or Slug for the repression of* CDH1* and EMT. Although the amounts of detailed Slug studies are fewer than those for Snail, the phosphorylation-mediated transcriptional regulation, stability, and degradation of Slug are described in the following section.

### 3.2. Regulation of Transcriptional Activity by Posttranslational Modification

The transcriptional repressor activity of Slug is supported by PAK4- and ERK2-mediated phosphorylation. A recent study revealed that PAK4 phosphorylates Slug at residues S158 and S254, which increases its transcriptional activity as a repressor of the* CDH1* promoter (Table 2) [31]. A phosphomimetic mutant of Slug at two residues suppressed the expression of* CDH1*, indicating that the phosphorylation of Slug at residues S158 and S254 is important for the regulation of its transcriptional activity. In addition to PAK4, ERK2 also activated Slug as a transcriptional regulator through the phosphorylation of residue S87; however, this phosphorylation does not regulate its stability or nuclear localization [32]. The phosphorylation of Slug at residue S87 is essential for its ability to induce vimentin or Axl expression for EMT, although this is a separate mechanism from the role of Slug as a transcriptional repressor of* CDH1*. Additional phosphorylation sites of Slug were detected at residues S4 and S88 based on an* in vivo* analysis, and the phosphorylation of Slug at residue S4 was found to increase the transcriptional repression of* CDH1* expression [66].

### 3.3. Degradation of Slug by Posttranslational Modification

Like Snail, the stability of Slug is regulated by GSK3*β*-mediated phosphorylation through CHIP (carboxyl terminus of Hsc70-interacting protein) at residues S92, S96, S100, and S104 (Table 2) [34, 36]. The phosphorylation of Slug by GSK3*β* provides the recognition sites of *β*-TRCP-mediated ubiquitination and proteasomal degradation. The activity of GSK3*β* limits the intracellular concentration of Slug, thus modulating its turnover by direct phosphorylation. Nondegradable Slug promotes cell migration, invasion, and cancer metastasis of lung adenocarcinoma [34]. Non-phospho-mimetic Slug at residues S92 and S96 was found to inhibit the degradation of Slug, while non-phospho-mimetic Slug at residues S100 and S104 was found to accumulate in the nucleus. Thus, the phosphorylation of Slug at residues S92 and S96 negatively affects its stability, while the phosphorylation of residues S100 and S104 affects its cytosolic localization and stability [36]. This indicates that GSK3*β*-mediated phosphorylation had a negative impact on* CDH1* repression. During the cell cycle of cancer cells during cancer progression, Slug is also phosphorylated by cyclin E/ CDK2, which promotes its proteasomal degradation at the G1/S phase transition [33].

### 3.4. Stabilization of Slug by Posttranslational Modification

In terms of posttranslational modifications, the stability of proteins is regulated by the balance between prodegradative modifications and defensive modifications against the degradation. In the case of Slug, PAK-mediated phosphorylation stabilizes Slug protein. PAK4 phosphorylates Slug at residues S158 and S254 for its stabilization by blocking its ubiquitination after GSK3*β*-mediated phosphorylation [31]. The expression of non-phospho-mimetics upregulates the transcriptional expression of* CDH*1 and reduces EMT, due to the reduced stability of Slug. Thus, PAK4-mediated phosphorylation of Slug at residues S158 and S254 is important to maintain the stability and its transcriptional repression activity (Table 2) [31]. Based on the phosphorylation of Snail by PAK1, Thaper and his colleagues observed whether PAK1 phosphorylates Slug around residue S246 of Snail. The kinase assay showed that PAK1 also phosphorylates Slug at residues S247, S251, and S254 [37]. The activation of PAK1 is dependent on the activation of Lyn tyrosine kinase, which triggers the phosphorylation of Slug and, therefore, its stabilization. Since the nuclear localization and stability of Slug is dependent on the activity of Lyn, PAK1 and Lyn kinases are positive regulators of Slug in EMT. In addition to phosphorylation, deacetylation modifications induce the stabilization of Slug. Deacetylation of Slug at residues K8 and K116 by SIRT2 prevents the degradation of Slug, which in turn increases its stability. SIRT2-mediated deacetylation of Slug is sufficient to increase the protein half-life and activity of Slug via stabilization [38].

Although Slug has been widely studied, these studies have been limited to breast cancer due to its constitutive overexpression in aggressive breast cancer. However, this protein needs to be studied in a range of different cancer systems to determine its unique function compared to Snail. Further studies could be used to demonstrate the importance of Slug in EMT. Furthermore, the posttranslational modification of Slug may account for its functional flexibility in EMT.

## 4. ZEB1/2

### 4.1. Function and Structure of ZEB1/2

The zinc finger E-box binding homeobox (ZEB) family is composed of ZEB1 (also known as TCF8, or deltaEF1) and ZEB2 (also named SIP1). They are transcription factors characterized by the presence of a zinc finger DNA-binding domain, which is required for transcriptional activity [67, 68]. ZEB1 was first identified as a repressor of the delta 1-crystallin enhancer and expressed in mesodermal tissues, suggesting that ZEB1 is involved in embryogenesis and development [69]. Loss of function experiments with ZEB1 demonstrated that the developmental defects related with mesenchymal-epithelial transition showed E-cadherin expression and vimentin depletion in embryonic tissues [70], suggesting that ZEB1 could have an important function in EMT. In several human cancer cell lines, the expression of ZEB1 induces EMT, as well as cancer cell invasion and metastasis [7, 71, 72]. ZEB expression is triggered by diverse growth factors and signaling pathways, including TGF-*β*, Wnt, and Notch signaling [73]. ZEB1 has two zinc finger domains in the N- and C-terminals, both of which bind to the E-box motif of the* CDH1* promoter region. During EMT, ZEB1 and ZEB2 suppressed the expression of* CDH1* directly through the recruitment of the corepressors CtBP and HDAC1 [74]. These corepressors bind to the protein binding domains of ZEB1, which are a CtBP interaction domain (CID) at the C-terminus, a Smad interaction domain (SID), and a homeodomain (HD) in the middle and a CAF/p300 binding domain (CBD) at N-terminus of ZEB1 [75] (Figure 1(b)). The recruitment of corepressors to specific binding sites allows ZEB1 to function specifically as a transcription factor of EMT. ZEB2 has similar structure domains with two zinc finger domains, an N-terminal CBD, Smad-binding domain (SBD), and HD and a C-terminal CID [76]. The function and stability of ZEB1 are regulated by the posttranscriptional modification of miRNA-200 and miRNA-203 [77]. ZEB1 needs to be studied further in order to fully elucidate the correlation between the posttranslational modification of this protein and its functions.

### 4.2. Regulation of Activity, Stability, and Location of ZEB by Posttranslational Modification in EMT

The ZEB posttranslational modification studies are relatively fewer than those of other transcription factors, including Snail, Slug, and Twist. Interestingly, the majority of the results related with the posttranslational modification of ZEB1/2 are found that ZEB1/2 function is negatively regulated. The transcriptional repression of ZEB1 is disrupted by phosphorylation and sumoylation, which suppresses its transition from epithelial-to-mesenchymal characteristics.

First, the phosphorylation of ZEB1 at residues T851, S852, and S853 by PKC inhibits the transcriptional activity induced by IGF-1 treatment [76]. Under IGF-1 treatment, the ERK1/2 pathway is activated and phosphorylates ZEB1 at T867. ERK1/2-mediated phosphorylation of ZEB1 disrupts its nuclear localization by IGF-1 treatment, which consequently disrupts its transcriptional activity [76]. In Llorens's study, two NLS were detected at two different regions, the first at amino acids 111–241 prior to the zinc finger domain and the second at amino acids 869–875 after the phosphorylation site at residue T867 [76]. Thus, it is plausible that the phosphorylation of residue T867 directly disrupts the interaction between its NLS regions and importin, although more evidence is needed.

Second, the sumoylation of ZEB1/2 is triggered by the polycomb protein Pc2, which reduces the transcriptional activity [78]. ZEB1 is sumoylated at residues K347 and K774, while ZEB2 is sumoylated at residues K391 and K866, which relieve E-cadherin repression [78]. As a mechanism of relieving E-cadherin repression, the sumoylation of ZEB2 at residue K866 disrupts the recruitment of corepressor CtBP, since this site is near a CtBP-binding motif. Thereby, Pc2-mediated sumoylation reduces the recruitment of CtBP1 for transcriptional repression [78].

Although ERK1/2 and PKC are negative regulators of ZEB1 in EMT, DNA damage sensing kinase ATM positively regulates ZEB1 function in response to DNA damage. ZEB1 function has been investigated in relation to radioresistance since ZEB1 is highly expressed in radioresistant-cancer cells [75]. ATM phosphorylates ZEB1 at residue S585 in response to DNA damage, which accelerates its interaction with USP7, a deubiquitinase, thus increasing the stability of ZEB1 [75], which may contribute to radioresistance in cancer cells.

## 5. Twist1/2

### 5.1. Function and Structure of Twist1/2

As an indirect repressor of the* CDH1* promoter, the basic helix-loop-helix (bHLH) transcription factor Twist1/2 is well-characterized compared to other indirect repressors, such as FOXC2 [13], SIX1 [14], and bHLH factor E2.2 [15]. Twist is evolutionarily conserved in species ranging from the fruit fly to humans. In mammals, two types of Twist, Twist 1 and Twist 2, exist. Twist 1 was first detected in* Drosophila* as an essential gene for early embryo development. Twist 1 has a DNA-binding basic region (amino acids 109–121), a bHLH domain (amino acids 122-163), and a Twist WR domain (amino acids 182-202) for transcriptional activity [79] (Figure 1(c)). The two Twists share a similarity of 100% in the C-terminal Twist box, 95% in the bHLH domain, and 54% in the N-terminal region [80]. The major structural differences of Twist 1 and Twist 2 are in the protein size and the N-terminal domains. Twist 1 has two glycine-rich regions (GRR) with 202 amino acids, while Twist 2 does not have a glycine-rich region and has 160 amino acids [80]. The transcriptional activity of Twist is activated by the dimerization of the Twist WR domain, which recognizes a unique tandem E-box motif in the proximal region of the promoters of target genes [81, 82]. Its binding efficiency to the E-box motif of the target gene's promoter is much better when Twist forms a heterodimer with another helix-loop-helix domain containing transcription factors [80–82]. Once Twist binds to these E-boxes, it can transcriptionally repress the expression of E-cadherin and consequently disrupts cell adhesion for the cell dissemination from the primary tumor site and subsequent metastasis [83]. Clinically, Twist functions as a prometastatic factor whose expression is associated with a poor clinical prognosis in several types of cancer [84, 85].

### 5.2. Regulation of Dimerization and Transcriptional Activity by Posttranslational Modification

Since Twist 1 forms a heterodimer or homodimer for transcriptional activation, the effects of Twist 1 on phosphorylation have been investigated in terms of its preference between heterodimer and homodimer formation. According to Wang and colleagues, Aurora A kinase directly phosphorylates Twist 1 at residues S123, T148, and S184 (Table 3) [39]. These modifications result in an increased transcriptional activity and inhibited ubiquitylation and favor homodimerization over heterodimerization with E12 and Hand2 [39]. Notably, p-S123 and p-T148 may regulate its partner binding, while p-S184 may affect its transcriptional activity directly. In addition, the choice of partner and the DNA-binding capacity of Twist 1 may be affected by the phosphorylation of residues T125 and S127 of the Thr-Gln-Ser (TQS) motif in the bHLH domain [40]. During development, PKA regulates the partner preference of Twist 1 and its DNA-binding capacity by phosphorylating residues T125 and S127 at a highly conserved TQS motif. The phosphorylation of Twist 1 at the TQS motif induces Twist 1–E12 heterodimerization [40], suggesting that Twist 1 phosphorylation at residues T125 and S127 determines its dimeric partner choice, which affects the induced metastatic activity of Twist 1.

As a positive regulatory phosphorylation of transcriptional activity, Akt-mediated phosphorylation of Twist 1 at residue S42 modulates its transcriptional target TGF-*β*2, resulting in prometastasis [41, 42]. Furthermore, hyaluronic acid binding to CD44-induced c-Src activation promotes Twist 1 phosphorylation, which increases its transcriptional activity and nuclear localization in breast cancer [86]. Despite this, the exact phosphorylation sites and preferences of dimerization have not been fully elucidated. Methylation also affects the transcriptional activity of Twist 1. The methylation of Twist 1 at residue R34 by PRMT1 is important for the transcriptional activity of E-cadherin repression [46].

### 5.3. Degradation of Twist by Posttranslational Modification

The levels of Twist are regulated by ubiquitination, which leads to its degradation, wherein certain types of signaling trigger its degradation in order to regulate EMT process. Twist is also a ubiquitin substrate of *β*-TRCP, the adaptor subunit of the E3 ligase complex. IKK*β* phosphorylates Twist at residues T125 and S127, where it is phosphorylated by PKA, which determines its prometastatic properties depending on its dimeric partner (Table 3) [40, 43]. However, the phosphorylation of Twist by IKK*β* induces its cytoplasmic translocation to accelerate *β*-TRCP-mediated destruction, suggesting that IKK*β* is a negative regulator of EMT via the degradation of Twist 1. Even if the same residues in Twist 1 are phosphorylated, depending on the cellular context, different signaling pathways can be triggered, which determine the proteins interacting in the Twist 1-kinase complex and, consequently, on the effect to be exerted. However, the reason for these different patterns outcomes is yet to be elucidated. Another kinase, AKT1, also regulates the degradation of Twist 1 by the phosphorylation of residues T121 and S123. Although AKT1 phosphorylates Twist 1 at three sites, S42, T121, and S123, the phosphorylation of two residues, T121 and S123, alone increased its degradation via *β*-TRCP-mediated ubiquitination, leading to the inhibition of EMT [42]. Although the phosphorylation of residue S42 increases the transcriptional activity and enhances EMT, AKT1 may exert dual functions in Twist 1 and EMT. The findings of these studies are proof of the flexible characteristics of Twist 1 in regulating the transition from epithelial-to-mesenchymal cells.

### 5.4. Stabilization by Posttranslational Modification

The stability of Twist 1, a prometastatic transcription factor, is directly related to cancer invasiveness and metastasis. The oncogenesis signaling pathway also contributes to the stability of Twist 1 for metastasis. Activated MAPKs, including p38, c-Jun N-terminal kinase, and extracellular signal-regulated kinase 1/2 by TGF-*β* treatment, markedly phosphorylate Twist 1 at residue S68. This phosphorylation results in increased levels of Twist 1 protein without changing its mRNA levels, while the inhibition of MAPK activities reduces the phosphorylation of Twist 1 and therefore its protein levels. Thus, MAPKs are a positive regulator of Twist 1 via its stabilization for metastasis (Table 3) [45]. The stability of Twist is also regulated at the posttranslational level in head and neck squamous cell carcinomas through casein kinase 2 phosphorylation of Twist at residues S18 and S20 [44].

Aurora A kinase promotes the transcriptional activation of Twist 1, as mentioned previously [40]. In addition to this function, the phosphorylation of Twist at residues S123, T148, and S184 by Aurora A kinase also increases the levels of Twist 1 protein, possibly by inhibiting its ubiquitination [40]. The transcriptional activity of Twist depends on its dimeric partner and results from the phosphorylation of Twist 1 at residues S123, T148, and S184. As such, the stability of Twist 1 is directly associated with its functional activity in EMT, invasiveness, and cell plasticity.

## 6. Conclusions

The phosphorylation of EMT transcription factors demonstrates the flexibility of their ability to regulate the progression of EMT for cell plasticity. The reports on Twist 1 demonstrate that one kinase can phosphorylate several residues with different functions. On the other hand, the phosphorylation of the same residue triggered by different kinases can have different outcomes, depending on the cellular context of the signaling pathways and protein interacting networks. These patterns could be related to the ability of cancer cells to switch between epithelial traits for tumorigenic proliferation and the mesenchymal phenotype for invasion and migration. It is currently widely accepted that metastatic, distant tumors contain characteristics of both EMT and mesenchymal-epithelial transition (MET). However, the phenotypic consequences of having partially EMT and partially MET traits have not yet been elucidated. It is possible that EMT and MET phenotypes are mixed in metastatic cancer because, as in most EMT regulatory factors, there are no permanent genetic alterations, but rather transient changes. As such, it would appear that posttranslational modifications and phosphorylation in particular are an appropriate tool to modify the epithelial and mesenchymal cellular events in a flexible and reversible manner. A small change like phosphorylation has the ability to change the entire cellular context since it can activate streams of cellular events and eventually influence the whole biological system of an organism.

## Figures and Tables

**Table 1:** The posttranslational modification sites of Snail for EMT.

Residues-modification	Kinase/Enzyme	Function	Cell Type	Reference
*Phosphorylation*				

S11-p	PKA	TF activity	HEK 293T, MDCK	[19]
		Stabilization		
	PKD1	Nuclear export of snail (Destabilization)	Breast epithelial cell	[20, 21]
		Suppression of EMT		

S82-p	ERK2	Nuclear accumulation	Breast cancer	[22]

S92-p	CK2	TF activity	HEK 293T, MDCK	[19]
		Stabilization		

S96/S100/	GSK3*β*	Degradation	prostate cancer	[23, 24]
S104/S107-p			Breast cancer	

S104/S107-p	CK1	Degradation	prostate cancer	[24]

S104-p	ERK2	Nuclear accumulation	Breast cancer	[22]

T203-p	Lats2	Nuclear accumulation		[25]
		Stabilization		

S246-p	PAK1	Nuclear accumulation	Breast cancer	[26, 27]
		TF activity	Non-small lung cancer	
		Stabilization		
	PI3K	Nuclear accumulation	Bladder cancer	[28]

*Other posttranslational modifications*				

S112-gl	O-GlcNAc	Stabilization		[29]

K206/K234/	A20	Monoubiquitylation	Breast cancer	[30]
K235-ub		Stabilization		

**Table 2:** The posttranslational modification sites of Slug for EMT.

Residues-modification	Kinase/Enzyme	Function	Cancer Type	Reference
*Phosphorylation*				

S158/S254-p	PAK4	Stabilization	Prostate	[31]
		TF activity		

S87/S104-p	ERK2	TF activity	Breast	[32]

S54/S104-p	Cyclin	Degradation	Lung	[33]
	E/CDK2			

S92/S96/S100/S104-p	GSK-3*β*	Degradation	Non-small cell lung	[34]
		Degradation	Breast	[35]
		Degradation	Liver, breast	[36]

S247/S251/S254-p	PAK1	Stabilization	Breast, bladder	[37]

*Acetylation*				

K8/K116-Ac	Deacetylase	Stabilization	Basal-like	[38]
	SIRT2		breast	

**Table 3:** The posttranslational modification sites of Twist 1 for EMT.

Sites	Kinase/Enzyme	Function	Reference
*Phosphorylation*			

S123/T148-p	AURKA	TF activity (Partner choice)	[39]
		Stabilization	
		Homodimerization	

S184-p	AURKA	TF activity (Direct)	[39]
		Stabilization	

T125/S127-p	PKA	TF activity (Partner choice)	[40]
		Heterodimerization	

S42-p	AKT1	TF activity	[41]

T121/S123-p	AKT1	Degradation	[42]

T125/S127-p	IKK*β*	Degradation	[43]
		Cytoplasm translocalization	

S18/S20-p	CK2	Stabilization	[44]

S68-p	P38, JNK, ERK1/2	Stabilization	[45]

*Methylation*			

R34-Me	PRMT1	E-cadherin repression	[46]
